# Biochemical effects of personalized clinical interventions on hepatorenal function, immune response, and inflammatory markers in patients with infections

**DOI:** 10.5937/jomb0-62921

**Published:** 2026-03-17

**Authors:** Ying Li, Ying Zhou, Jie Zhang, Yi Li, Xin Liu

**Affiliations:** 1 PeKing University People's Hospital, Department of Infectious Diseases, Beijing, China; 2 PeKing University People's Hospital, Department of Emergency, Beijing, China

**Keywords:** personalized intervention, infection, hepatorenal biomarkers, immune function, inflammatory cytokines, personalizovana intervencija, infekcija, hepatorenalni biomarkeri, imunološka funkcija, inflamatorni citokini

## Abstract

**Background:**

Hepatic and renal dysfunction, immune imbalance, and excessive inflammatory activation are common complications in postoperative infections. This study evaluated the biochemical effects of personalized clinical interventions on hepatorenal function, immune responses, and inflammatory cytokines in infected patients.

**Methods:**

A total of 110 patients with postoperative infections admitted between January 2023 and January 2025 were randomly assigned to an experimental group or a control group (n = 55 each). The control group received standard clinical care, while the experimental group underwent additional individualized intervention measures. Serum hepatic (AST, ALT) and renal (BUN, SCr, UA) biochemical markers, T-lymphocyte subsets (CD3+, CD4+, CD8+), inflammatory factors (IL-6, CRP TNF-a), and SF-36 quality-of-life scores were assessed before and after intervention. Adverse events were recorded.

**Results:**

Post-intervention, AST and ALT levels were significantly lower in the experimental group compared with the control group (P &lt; 0.05). BUN, SCr, and UA were also significantly reduced after individualized intervention (P &lt; 0.05). The experimental group demonstrated higher CD3+ and CD4+ levels and lower CD8+ levels than the control group (P &lt; 0.05). Levels of IL-6, CRP and TNF-a were markedly decreased in the experimental group (P &lt; 0.05). Quality-of-life scores across all domains were significantly higher following individualized intervention (P &lt; 0.05). The incidence of adverse reactions was notably lower in the experimental group (5.45% vs. 20.00%; c2 = 4.010, P = 0.045).

**Conclusions:**

Personalized clinical interventions significantly improved hepatic and renal biochemical indices, enhanced immune function, and reduced systemic inflammatory cytokines in patients with postoperative infections. These findings support the integration of individualized strategies into infection management to optimize biochemical and clinical outcomes.

## Abstract (sr)

**Uvod:**

Hepatična i bubrežna disfunkcija, imuni disbalans i prekomerna inflamatorna aktivacija su česte komplikacije kod postoperativnih infekcija. Ova studija je procenila biohemijske efekte personalizovanih kliničkih intervencija na hepatorenalnu funkciju, imune odgovore i inflamatorne citokine kod inficiranih pacijenata.

**Metode:**

Ukupno 110 pacijenata sa postoperativnim infekcijama primljenih između januara 2023. i januara 2025. godine nasumično su raspoređeni u eksperimentalnu ili kontrolnu grupu (n = 55 u svakoj grupi). Kontrolna grupa je primala standardnu kliničku negu, dok je eksperimentalna grupa podvrgnuta dodatnim individualizovanim intervencijama. Serumski hepatični (AST, ALT) i bubrežni (BUN, SCr, UA) biohemijski markeri, podskupovi T-limfocita (CD3+, CD4+, CD8+), inflamatorni faktori (IL-6, CRP TNF-a) i SF-36 rezultati kvaliteta života procenjeni su pre i posle intervencije. Zabeleženi su neželjeni događaji.

**Rezultati:**

Nakon intervencije, nivoi AST i ALT bili su značajno niži u eksperimentalnoj grupi u poređenju sa kontrolnom grupom (P &lt; 0,05). BUN, SCr i UA su takođe značajno smanjeni nakon individualizovane intervencije (P &lt; 0,05). Eksperimentalna grupa je pokazala više nivoe CD3+ i CD4+ i niže nivoe CD8+ nego kontrolna grupa (P &lt; 0,05). Nivoi IL-6, CRP i TNF-a bili su značajno smanjeni u eksperimentalnoj grupi (P &lt; 0,05). Rezultati kvaliteta života u svim domenima bili su značajno viši nakon individualizovane intervencije (P &lt; 0,05). Učestalost neželjenih reakcija bila je znatno niža u eksperimentalnoj grupi (5,45% naspram 20,00%; c2 = 4,010, P = 0,045).

**Zaključak:**

Personalizovane kliničke intervencije značajno su poboljšale biohemijske indekse jetre i bubrega, poboljšale imunološku funkciju i smanjile sistemske inflamatorne citokine kod pacijenata sa postoperativnim infekcijama. Ovi nalazi podržavaju integraciju individualizovanih strategija u upravljanje infekcijama kako bi se optimizovali biohemijski i klinički ishodi.

## Introduction

Infectious diseases remain a major clinical burden and can profoundly impair patients' health and quality of life. During the onset and progression of infection, disturbances in hepatorenal function and immune homeostasis play central roles in determining disease severity and clinical outcomes [1]. The liver and kidneys are key metabolic and detoxification organs whose functional integrity is particularly susceptible to the deleterious effects of pathogens and pathogen-derived toxins. For example, endotoxins produced during bacterial infections can activate innate immune pathways, drive excessive inflammatory responses, and consequently trigger immune-mediated hepatic and renal injury. Likewise, viral pathogens may directly invade hepatocytes and renal tubular epithelial cells, thereby compromising their physiological functions and metabolic capacity [2]. Impaired hepatic metabolism can, in turn, diminish the clearance of both pathogens and therapeutic agents, increasing the risk of adverse drug reactions. Similarly, renal dysfunction reduces the excretion of metabolic waste products and toxins, further aggravating systemic biochemical imbalances.

The immune response constitutes the primary defense against pathogenic invasion. When properly regulated, the immune system can efficiently recognize and eliminate pathogens; however, immune dysregulation—whether in the form of hyperactivation or suppression—can result in tissue injury or inadequate pathogen control [3]. Consequently, preserving hepatorenal stability and maintaining balanced immune activity are critical for enhancing treatment efficacy and improving clinical prognosis in patients with infectious diseases [4].

Conventional clinical management strategies often rely on standardized care processes that may not fully address interindividual variability in disease manifestation or host responses. Personalized clinical interventions, by contrast, emphasize individualized assessment and dynamic adjustment of supportive measures based on factors such as age, comorbidities, infection type, physical condition, and psychological state [5]. Such individualized approaches— through targeted monitoring of hepatorenal biochemical indices, careful medication optimization, tailored nutritional strategies, and structured psychological support—have the potential to modulate immune and inflammatory responses, reduce organ burden, and promote recovery [6].

Given the central importance of biochemical markers in evaluating organ injury and immune-inflammatory status, this study investigates the biochemical effects of personalized clinical interventions on hepatorenal function, immune responses, and inflammatory cytokines in patients with infections.

## Materials and methods

### General information

A total of 110 patients who developed postoperative infections in the emergency department of our hospital between January 2023 and January 2025 were enrolled. Using a random number table method, patients were assigned to either the experimental group or the control group, with 55 patients in each group. Baseline demographic and clinical characteristics showed no significant differences between groups (P > 0.05), as summarized in Table 1.

**Table 1:** Comparison of baseline data between the two groups (x̄±s, n(%)).

Group	n	Gender (male/female)	Age (years)	Duration of infection (days)
Experimental group	55	32/23	50.30 ± 6.10	10.81 ± 1.69
Control group	55	30/25	51.22 ± 5.88	1.24
t/χ^2^		0.148	0.805	1.24
P		0.7	0.423	0.218

### Inclusion and exclusion criteria

### Inclusion criteria:

1. Diagnosis of infection confirmed by clinical manifestations and laboratory examinations (e.g., routine blood tests, pathogen cultures);
2. Age 18-75 years;
3. Intact cognitive function with the ability to cooperate with interventions;
4. Provision of informed consent by patients and their family members.

### Exclusion criteria:

1. Presence of severe psychiatric disorders such as schizophrenia or major depressive disorder;
2. Pregnancy or breastfeeding;
3. Severe immunodeficiency;
4. Diagnosis of malignant tumors.

### Intervention procedures

### Control group

Patients in the control group received routine clinical care, which included regular monitoring of vital signs (e.g., temperature, blood pressure) and close observation of symptom changes. Standardized disinfection and isolation procedures were implemented to prevent cross-infection. Medications were administered in accordance with physician prescriptions, ensuring accuracy and timely delivery while monitoring for drug-related adverse reactions. Nutritional, easily digestible meals were provided, and psychological support was offered as needed. Patients were also guided to maintain appropriate rest-activity balance to support recovery.

### Experimental Group

Patients in the experimental group received personalized clinical interventions in addition to routine care. These individualized measures included the following components:

### (1) Comprehensive assessment

A structured assessment was conducted covering demographic characteristics (age, gender, occupation), infection type and severity, infection duration, medication and allergy history, and psychological status (e.g., anxiety, fear). Lifestyle habits—including dietary preferences and activity patterns—were evaluated to guide personalized intervention planning.

### (2) Development of personalized care plans

Based on assessment findings, individualized management plans were formulated. Examples included temperature-control measures for febrile patients (e.g., physical cooling or antipyretics under physician instruction) and respiratory techniques for patients with respiratory tract infections (e.g., breathing exercises, sputum-expulsion training). Dietary guidance was tailored according to comorbidities, such as low-sugar, nutrient-dense diets for diabetic patients. Psychological support strategies were customized to patient personality and emotional state.

### (3) Implementation of interventions

Interventions were implemented strictly in accordance with the individualized care plan. Vital signs - including temperature, blood pressure, and heart rate - were closely monitored, documented, and promptly reported in the event of any abnormal changes. Medications were administered on schedule to ensure optimal therapeutic efficacy. Patients were assisted in maintaining personal hygiene and provided with a quiet, comfortable environment. Regular communication and emotional support were offered to alleviate anxiety and enhance adherence to treatment.

### (4) Dynamic adjustment of care plans

Interventions were continuously reviewed and adjusted based on clinical changes and patient responses. If symptoms worsened or new complications developed, corresponding measures - such as enhanced isolation, medication adjustment, or modified psychological support - were promptly implemented.

### (5) Targeted health education

Health education was tailored to patients' educational background and comprehension. Information regarding infection-related knowledge, treatment procedures, medication use, dietary recommendations, and exercise strategies was explained clearly to improve self-management capacity and reduce reinfection risk.

*Both groups received their respective interventions continuously for a duration of three months.*

### Observation indicators

### (1) Hepatic biochemical markers:

Fasting venous blood samples (5 mL) were collected before and after the intervention. Serum aspartate aminotransferase (AST) and alanine aminotransferase (ALT) were measured using enzyme-linked *immunoassay*.

### (2) Renal biochemical markers:

Serum blood urea nitrogen (BUN), serum creatinine (SCr), and uric acid (UA) levels were assessed using an automated biochemical analyzer.

### (3) Immune function indicators:

Peripheral blood T-lymphocyte subsets (CD3^+^, CD4^+^, CD8^+^) were measured using flow cytometry.

### (4) Inflammatory cytokines:

Serum interleukin-6 (IL-6), C-reactive protein (CRP), and tumor necrosis factor-α (TNF-α) concentrations were quantified using enzyme-linked immunosorbent assay (ELISA).

### (5) Quality-of-life assessment:

Quality of life was evaluated before and after intervention using the SF-36 scale, including physical function, psychological function, health status, and social function. Higher scores indicated better quality of life.

### (6) Adverse reactions:

Adverse events—including persistent fever, nau-sea/vomiting, abdominal distension/diarrhea, and constipation—were documented throughout the study period.

### Statistical analysis

Statistical analyses were performed using SPSS version 26.0 (IBM, Armonk, NY, USA). Categorical variables were expressed as frequencies and percentages [n (%)] and compared using the χ^2^ test. Continuous variables were reported as mean ± standard deviation (x̄±s) and compared using independent-samples or paired t-tests, as appropriate. A twosided P-value < 0.05 was considered statistically significant.

## Results

### Comparison of liver function indicators before and after intervention

Following the intervention period, serum AST and ALT levels in the experimental group were significantly lower than those in the control group ( \ (P < 0.05 \ )), indicating a greater improvement in hepatic biochemical function. The detailed results are presented in Table 2.

**Table 2:** Comparison of liver function indicators before and after intervention between the two groups (x̄±s, U/L). *Note: Compared with before intervention, P < 0.05.

Group	n	AST			
		Before intervention	After intervention	Before intervention	After intervention
Experimental group	55	66.89±5.03	32.60±3.23*	120.87±14.21	25.87±2.94*
Control group	55	67.61±5.61	47.73±4.76*	121.76±14.42	46.67±4.10*
t		0.709	19.506	0.326	30.575
P		0.48	<0.001	0.745	<0.001

### Comparison of renal function indicators before and after intervention

Post-intervention measurements showed that BUN, SCr, and UA levels in the experimental group were significantly lower than those in the control group ( \ (P < 0.05 \ ) ). These results indicate superior renal biochemical recovery in patients who received personalized clinical interventions. The relevant data are summarized in Table 3.

**Table 3:** Comparison of renal function indicators before and after intervention between the two groups (x̄±s). *Note: Compared with before intervention, P < 0.05.

Group	n	BUN (mmol/L)		SCr (μmol/L)		UA (μmol/L)	
		Before	After	Before	After	Before	After
Experimental<br>group	55	45.37±4.85	14.64±1.24*	455.48±35.73	240.15±14.61*	515.34±25.12	326.87±14.51*
Control<br>group	55	45.53±4.82	18.76±1.75*	456.36±34.76	265.13±21.02*	516.37±25.07	364.63±20.08*
t		0.174	18.76±1.75*	0.131	7.237	0.215	11.304
P		0.862	<0.001	0.896	<0.001	0.83	<0.001

### Comparison of immune function indicators before and after intervention

After treatment, the experimental group exhibited significantly higher levels of CD3^+^ and CD4^+^ T-lymphocyte subsets, along with significantly lower CD8^+^ levels, compared with the control group ( \ ( P < 0.05 \ ) ). These findings indicate an improved immunological profile associated with the individualized intervention strategy. The corresponding values are presented in Table 4.

**Table 4:** Comparison of immune function indicators before and after intervention between the two groups (x̄±s, %).

Group	n						
		Before	After	Before	After	Before	After
Experimental<br>group	55	40.64±4.23	60.04±5.61*	30.22±3.18	38.07±4.11*	35.81±3.17	27.63±2.15*
Control<br>group	55	40.51±4.12	54.53±4.68*	30.16±3.24	34.59±3.82*	35.53±3.03	31.23±2.72*
t		0.163	5.593	0.098	4.6	0.474	7.7
P		0.871	<0.001	0.922	<0.001	0.636	<0.001

### Comparison of inflammatory factor levels before and after intervention

Serum concentrations of IL-6, CRP and TNF-α were markedly lower in the experimental group than in the control group following intervention (P < 0.05). This demonstrates a more pronounced reduction in systemic inflammation among patients receiving personalized care. Detailed data are provided in Table 5.

**Table 5:** Comparison of inflammatory factor levels before and after intervention between the two groups (x̄±s). *Note: Compared with before intervention, P < 0.05.

Group	n	IL-6 (ng/L)		CRP (mg/L)		TNF-a (ng/mL)	
		Before	After	Before	After	Before	After
Experimental<br>group	55	82.05±8.30	32.82±3.43*	32.12±3.25	18.02±1.56*	25.45±2.21	8.68±1.34*
Control<br>group	55	82.12±8.62	48.16±4.99*	32.51±3.79	25.56±2.53*	25.27±2.16	13.35±1.69*
t		0.043	18.788	0.579	18.813	0.432	16.058
P		0.966	<0.001	0.564	<0.001	0.667	<0.001

### Comparison of quality-of-life scores before and after intervention

Scores for physical function, psychological function, overall health status, and social function were significantly higher in the experimental group than in the control group after the intervention ( \ ( P < 0.05 \ ) ). These findings indicate better multidimensional recovery in the personalized intervention group. The detailed results are presented in Table 6.

**Table 6:** Comparison of quality of life scores before and after intervention between the two groups (x̄±s, points). *Note: Compared with before intervention, P < 0.05.

Group	n	Physical Function		Psychological Function		Health Status		Social Function	
		Before	After	Before	After	Before	After	Before	After
Experimental<br>group	55	50.52±4.93	72.06±6.78*	50.24±4.28	73.88±6.44*	51.24±4.64	70.93±6.62*	52.50±4.63	70.30±6.42*
Control<br>group	55	50.29±4.75	63.23±5.25*	50.96±4.12	62.98±5.04*	51.13±4.67	64.79±5.78*	52.67±4.48	65.82±5.64*
t		0.249	7.637	0.899	9.885	0.124	5.181	0.196	3.888
P		0.804	<0.001	0.371	<0.001	0.902	<0.001	0.845	<0.001

### Comparison of adverse reaction rates

The incidence of adverse reactions was significantly lower in the experimental group (5.45%) than in the control group (20.00%) (χ^2^ = 4.010, P = 0.045), suggesting enhanced safety and tolerability of the individualized intervention protocol. Details are shown in Table 7.

**Table 7:** Comparison of adverse reaction rates between the two groups (n, (%)).

Group	n	Persistent Fever	Nausea/Vomiting	Abdominal<br>Distension/Diarrhea	Constipation	Total Incidence
Experimental<br>group	55	1 (1.82)	1 (1.82)	0 (0.00)	1 (1.82)	3 (5.45)
Control<br>group	55	3 (5.45)	3 (5.45)	2 (3.64)	3 (5.45)	11 (20.00)
χ^2^						4.01
P						0.045

## Discussion

Personalized clinical interventions have the potential to substantially improve outcomes in patients with infectious diseases by modulating key biochemical, immunological, and inflammatory pathways. Traditional care models emphasize standardized procedures for monitoring vital signs, administering medications, and providing basic psychological support. Although these approaches contribute to treatment accuracy and the prevention of complications [7][8], their uniform nature often fails to account for the considerable interindividual variability observed in infectious disease presentations. Consequently, subtle physiological changes may be overlooked, and generalized health education may not align with patients' cognitive levels or specific clinical needs, leading at times to incomplete understanding and suboptimal self-management [9][10][11].

In contrast, individualized clinical strategies offer distinct advantages through intensive monitoring, patient-specific planning, and dynamic adjustment of therapeutic measures. Tailored assessments enable the timely identification of changes in infection severity or organ function, thereby facilitating early treatment modifications and more effective coordination of care [12]. Furthermore, psychological interventions calibrated to patients' emotional resilience can reduce stress responses and enhance treatment adherence [13]. Targeted lifestyle and nutritional guidance further supports systemic recovery by addressing individual physiological demands [14].

The biochemical findings of this study provide objective evidence that personalized interventions modulate hepatorenal function more effectively than standard care. Serum AST and ALT levels were significantly lower in the experimental group, indicating attenuation of hepatocellular injury. This improvement may be attributable to optimized nutritional support that promotes hepatocyte repair, improved metabolic homeostasis, and a reduced inflammatory burden associated with lower stress responses. In addition, early detection of biochemical abnormalities and timely adjustment of pharmacotherapy likely contributed to enhanced hepatic protection.

Similarly, the significant reductions in BUN, SCr, and UA indicate more favorable renal biochemical recovery. Infection-related renal impairment is frequently linked to inflammatory cytokine release, ischemic stress, and drug-related nephrotoxicity [15]. The individualized approach likely mitigated these factors through closer surveillance of renal function, more cautious use of potentially nephrotoxic medications, and improved hydration and metabolic support, thereby preventing further renal burden and facilitating restoration of kidney function [16].

Immune parameters also improved markedly following individualized intervention. Increases in CD3^+^ and CD4^+^ levels, together with reductions in CD8^+^ levels, reflect enhanced T cell homeostasis and immune regulation. These changes may be driven by several synergistic factors, including improved nutritional intake (particularly micronutrients essential for lymphocyte proliferation), greater psychological stability that mitigates neuroendocrine stress, and increased physical activity that promotes immune cell circulation [17]. Collectively, these adaptations support a more effective immune response against ongoing infection.

Reductions in inflammatory cytokines provide additional biochemical evidence of therapeutic benefit. Elevated IL 6, CRF, and TNF α are hallmarks of systemic inflammatory activation and are closely associated with infection related organ injury, especially in the liver and kidneys [18]. Personalized interventions likely lowered these cytokine levels by enhancing stress regulation, optimizing nutritional status, and enabling timely therapeutic adjustments, thereby attenuating excessive inflammatory cascades [19]. This decreased inflammatory burden is consistent with the improvements in biochemical markers of organ function observed in this study.

In addition to objective biochemical improvements, quality-of-life indicators—including physical, psychological, overall health status, and social functioning scores - were significantly higher in the experimental group. These findings suggest that individualized strategies may facilitate not only organ-level recovery but also broader functional and psychosocial rehabilitation [20][21]. Improved patient engagement, enhanced self-management ability, and better symptom control likely contributed to these outcomes [22][23].

Furthermore, the markedly reduced incidence of adverse events in the experimental group indicates that individualized monitoring and timely therapeutic modifications help mitigate risks related to infection progression, pharmacotherapy, and metabolic stress. These safety advantages further reinforce the clinical value of tailored intervention models.

## Conclusion

In summary, personalized clinical interventions significantly improved hepatic and renal biochemical markers, enhanced immune function, and reduced systemic inflammatory cytokines in patients with infections. These biochemical improvements were accompanied by better quality-of-life outcomes and fewer adverse reactions, underscoring the comprehensive benefits of individualized management. However, the study is constrained by a relatively small sample size and short follow-up duration. Future research should therefore include larger cohorts and extended observation periods to clarify the long-term biochemical, immunological, and clinical effects of personalized interventions, as well as to further explore their underlying mechanisms. Overall, the findings support the adoption of individualized clinical strategies as an effective adjunct to standard infection management.

## Dodatak

### Conflict of interest statement

All the authors declare that they have no conflict of interest in this work.

### Contribution

Ying Li and Ying Zhou contributed equally to this work.
